# The Collective Interests of Nurses

**Published:** 1920-03-06

**Authors:** 


### THE MATRONS' AND SISTERS' DEPARTMENT.

### THE COLLECTIVE INTERESTS OF NURSES.

Comparatively few people realise to what extent the amazing new spirit of vitality in the nursing profession is due to the Local Centres of the College of Nursing. Before the College introduced the principle of decentralisation provincial nursing life can hardly be said to have existed. All the talents and energies were there ready for demonstration when the call for action came, but no sign of any corporate existence outside the walls of institutions was visible. The recent *Bulletin* of the College displays a charming picture of the things which nurses have found to do for themselves and their profession the moment they found their opportunity.

First, perhaps, in importance, may be mentioned the subject of State registration. The recognition of the profession by the State was rightly placed in the forefront of the programme in almost every locality. In some districts the efforts made were of a nature to carry conviction to any body of legislators who might profess doubt on the subject. For instance, at Edinburgh 3,000 Scottish nurses signed the Scottish Board's Representation, and nearly 5,000 communications were issued to doctors, Members of the Parliament and others. The success of the College in so quickly obtaining legislation is an object-lesson in combined effort.

All the Centres have done splendid spade-work for the development of the College and the spread of their own local organisation. When the short time that they have been in existence is remembered, it must be a matter of some astonishment that their labours have been so efficient and that the entirely new organising powers required should have been found ready to hand wherever required.

The general desire of members to have a meeting-ground of their own is one of the most strongly-marked features of these communities. Brighton is the first Centre to boast a fully-equipped residential club with bedrooms, but club-rooms and club-houses have been started in many towns, Edinburgh, Dublin, Aberdeen, Liverpool, etc., and in several towns affiliated to the Yorkshire Centre. Since these have so rapidly sprung into being it may be safely prophesied that before long every Local Centre will boast its own club. The satisfaction and comfort afforded to nurses by these means are incalculable.

The possession of club-rooms always brings with it a desire for recreation and social enjoyments. Nurses by no means desire to flee each other's society in their leisure hours. On the contrary, they assemble together with particular pleasure, and the promotion of social intercourse between members living in institutions and those who are engaged in private or municipal work is found to bring fresh interest and a sense of comradeship to both.

It is interesting to find that nurses meeting in conclave set to work invariably and at once to obtain further educational advantages. Post-graduate lectures are a common feature in every centre, and the spontaneous co-operation of the medical profession has never failed. A new current of professional eagerness has made itself felt in many parts of the country where nurses could not hitherto get into touch with recent medical and scientific discoveries, and the nurse "condemned to rust in the country" is well-nigh a being of the past.

The scholarship scheme of the College has been taken up with much enthusiasm, and we believe we are right in stating that every Centre has contributed towards it. At Derby a scholarship has been founded for midwifery training, open to members of the Centre, and at Dundee a special scholarship for training as a sister-tutor will shortly be available, on the initiative of Lady Cowdray, who has promised £100 towards it. The task of raising funds for the endowment of the College has been most generously shouldered in many Centres. That of East Lancashire has actually contributed £12,500 to this purpose. The Centres have generally secured the support of the Mayor and other influential persons in the effort to set their profession on a sound basis.

One of the most interesting results achieved by the Centres has been the promotion of unity among widely sundered branches of the profession. At Bristol, for instance, the Committee now embraces representatives from all the institutions employing nurses—military, general, special, and Poor-Law hospitals in the city, as well as in outlying districts from Taunton to Cheltenham. Local medical men and women have been enlisted, and headmistresses of schools have been drawn into sympathy with the College desire to raise the educational standard among probationers.

We have not space to dwell on the valuable work done in some Centres towards standardising the training in hospitals. But have we not said enough to show what nurses can accomplish in the spirit of unity?

